# Suicide Risk and Protective Factors in Online Support Forum Posts: Annotation Scheme Development and Validation Study

**DOI:** 10.2196/24471

**Published:** 2021-11-08

**Authors:** Stevie Chancellor, Steven A Sumner, Corinne David-Ferdon, Tahirah Ahmad, Munmun De Choudhury

**Affiliations:** 1 Department of Computer Science & Engineering University of Minnesota - Twin Cities Minneapolis, MN United States; 2 Office of Strategy and Innovation National Center for Injury Prevention and Control Centers for Disease Control and Prevention Atlanta, GA United States; 3 Division of Violence Prevention Centers for Disease Control and Prevention Atlanta, GA United States; 4 School of Interactive Computing Georgia Institute of Technology Atlanta, GA United States

**Keywords:** online communities, suicide crisis, construct validity, annotation scheme, Reddit, annotation

## Abstract

**Background:**

Online communities provide support for individuals looking for help with suicidal ideation and crisis. As community data are increasingly used to devise machine learning models to infer who might be at risk, there have been limited efforts to identify both risk and protective factors in web-based posts. These annotations can enrich and augment computational assessment approaches to identify appropriate intervention points, which are useful to public health professionals and suicide prevention researchers.

**Objective:**

This qualitative study aims to develop a valid and reliable annotation scheme for evaluating risk and protective factors for suicidal ideation in posts in suicide crisis forums.

**Methods:**

We designed a valid, reliable, and clinically grounded process for identifying risk and protective markers in social media data. This scheme draws on prior work on construct validity and the social sciences of measurement. We then applied the scheme to annotate 200 posts from r/SuicideWatch—a Reddit community focused on suicide crisis.

**Results:**

We documented our results on producing an annotation scheme that is consistent with leading public health information coding schemes for suicide and advances attention to protective factors. Our study showed high internal validity, and we have presented results that indicate that our approach is consistent with findings from prior work.

**Conclusions:**

Our work formalizes a framework that incorporates construct validity into the development of annotation schemes for suicide risk on social media. This study furthers the understanding of risk and protective factors expressed in social media data. This may help public health programming to prevent suicide and computational social science research and investigations that rely on the quality of labels for downstream machine learning tasks.

## Introduction

### Background

In the United States, suicide is a leading cause of death and a pressing public health concern [1,2]. Suicide rates have increased >30% over the past 20 years [2]. Suicide is preventable [1,3]—early identification and support of people at risk, such as those with suicidal ideation, is a proven strategy that can reduce suicide [3].

Digital communities and social networking platforms provide support to individuals who may be considering self-harm or suicide. Examples of such communities are r/SuicideWatch on Reddit [4-6], ReachOut [7-9], and TalkLife [10], which offer dynamic and organic support that assists those in need. For this aim, social media data have been harnessed as a naturalistic and unobtrusive source of information about how to improve suicide prevention [4,6,11-13].

A focus in recent research has been to conceptualize and quantify risk from web-based posts, thereby identifying who may be most in need of assistance. *Suicide risk* estimation assesses the likelihood that someone may attempt or die by suicide. For extracting measures of risk from these data, prior work has often conceptualized risk into categorical or ordinal groups—risky and not risky [4,6], a *stoplight* system of green, yellow, and red [7-9], or a 0 to 3 scale [13,14]. Categories are then mapped to training data for computational linguistic analysis and the development of machine learning models to quantify risk and potentially predict behavior [9,13,15-17].

Current quantifiable risk evaluations of suicidality map to a single perspective of evaluating risk, which focuses on aggregated notions of riskiness that may determine a response from a clinician. Instead of collapsing the notion of risk into a singular point, clinical and public health professionals instead often examine and track *risk factors* or attributes and characteristics that increase an individual’s likelihood of attempting suicide in the future [18]. Such health professionals also explore *protective factors* or characteristics and behaviors that decrease the probability of suicidal ideation, planning, or attempts [18,19]. These include both psychological factors, such as access to mental health care and treatment, and social factors, such as supportive family members. These factors are important as they provide resilience and a *buffer* against suicide [19,20]. Assessing both risk and protective factors provides a more nuanced and holistic view of the risk for suicide.

Labeling social media data for complex behaviors such as suicidality is simultaneously pervasive within research and challenging. Social media data do not include clinically validated signals of distress or diagnosis, and labels must therefore be generated. Agreeing on and applying these labels to data sets is difficult in part as the evaluation of mental health (especially for suicide risk) is more subjective and requires complex labeling schemes [21,22]. However, there are no current schemas that study risk and protective factors for suicidal ideation in social media data. Moreover, there are no practical guidelines on how to construct and validate annotation systems and schema for complex mental health behaviors in social media [23]. This is of critical concern given that recent research on mental health and social media has identified numerous challenges in how clinical and health signals are constructed, annotated, and verified in data sets [23-25]. The reliability and validity of these signals are essential for ensuring studies on social media data accurately measure what they claim to measure [26,27].

### Objective

To address this problem, we draw on the vocabulary and tools of construct validity measurement from the social sciences to formalize an annotation scheme. Measures of validity have a long and rich history in social sciences (under the name of measurement modeling) [27], computational linguistics [28,29], and psychometrics [30,31]. In this study, we focused on construct validity, or “making inferences from the sampling particulars of a study to the higher-order constructs they represent” [27,32]. In our case, this allows us to translate the higher-order clinical concept of *risk and protective factors* to those in social media. By using construct validity as an anchoring concept for our research, we aim to produce more accurate, representative, and reliable labels of risk and protective factors from digital text.

In this study, we provide the development process, a first validation, and results for a framework for operationalizing and testing clinical concepts via social media data. We do so by assessing the risk and protective factors of suicidal ideation in r/SuicideWatch, a Reddit community dedicated to social support during a suicide crisis event. A team of experts in social media, mental health, public health, and suicide worked collaboratively to develop this annotation scheme. We have provided detailed descriptions and procedures for iterative development and validation. Finally, we tested this approach on 200 posts from the community and discussed the initial results of our annotations and how they reflect on studying suicidal ideation in social media.

Our work provides a formalized approach for developing annotation data for suicide and social media data. We have discussed the implications of this research as they relate to the development of rigorous and validated frameworks for assessing mental health on the web. This work also considers downstream applications, such as expert annotation, training laypersons for generating training data in machine learning, closed coding for qualitative analysis or for grounded evaluation of machine learning model outcomes that assess suicide risk and buffers.

## Methods

### Our Approach to Labeling

Our research goals connect to the larger area of labeling data—a problem that applies across fields outside of computer science, such as linguistics [28] and psychometrics [30,31]. Given these considerations and our priorities for exploring construct validity through labeling [33], we designed a novel and iterative process for building an annotation scheme to evaluate suicide risk and protective factors in social media posts. We adopted the socioecological framework as the basis for labeling these factors. Initially focused on the sociological study of human development [34], socioecological models help conceptualize the dynamic and interrelated factors that influence outcomes in psychological behaviors [34], and in our case, suicidal ideation [2,35]. In addition to personal and individual factors, this model accounts for circumstances such as relationships, community, and social pressures that affect well-being.

Creating labels that accurately capture what is of interest is tied to *construct validity*, the degree to which a practical measure or label captures what theoretical concept it claims to measure [32]. Guided by the approach of Simms and Watson [31] to construct validity and psychometric instruments, we formalized an approach to annotation scheme development that aligns around two core questions for construct validity:

1. Is this annotation system needed, useful, and in alignment with prior work and expertise?
2. Is the annotation system reflective of the prior literature and able to be applied reliably across a research team?

**Figure 1:**
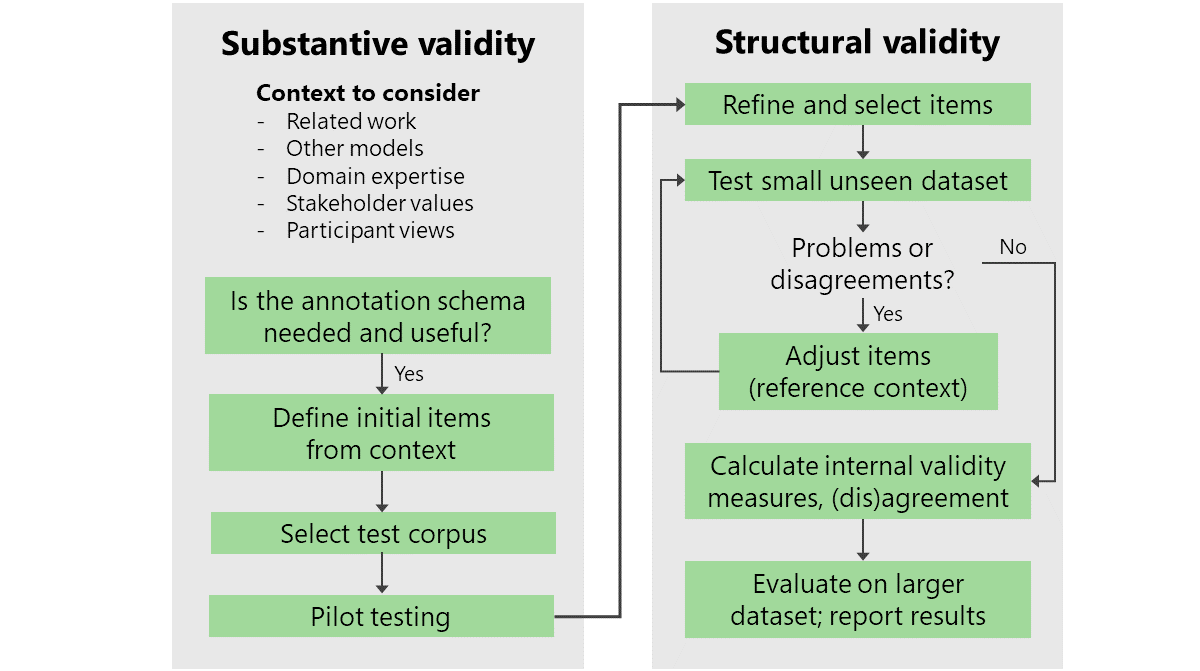
An overview of our annotation process.

We present an overview of this process in Figure 1. The first question approximates the process of *substantive validity,* which Simms and Watson [31] argue is “centered on the tasks of construct conceptualization and development of the initial item pool.“ We expand on the approach by Simms and Watson [31] for development to include crucial input from stakeholders and possibly participants for whom labels will be applied, adopting a stakeholder-driven and human-centered approach to social media data analysis [36]. The data set of interest and a pilot annotation scheme can then be developed.

Next comes the *structural validity* phase, where the scheme was tested against the construct in practical and measurable ways. We focused on two strategies for reaching consensus: small-scale testing and refinement of items and intergroup reliability testing. Raters apply the ratings to a random but small set of new examples from the social media corpus and engage in group discussions to adjust items and themes. Once consensus was reached, the raters independently annotated a larger batch of posts and recorded the metrics of interrater reliability to evaluate the consistency of the scheme.

In the following sections, we describe our application of this procedure to suicide risk and protective factors in social media data.

### Data Collection and Preparation

#### Source of Data

We used data from Reddit, a social media site organized into *subreddits,* individual communities organized around topic areas. We chose to study r/SuicideWatch, given its focus and interest from prior work [4,13,37,38] and ample text space for content (50,000 characters).

In June 2019, we gathered our data set from r/SuicideWatch (r/SW) from archived, public Reddit data through Google’s BigQuery data storage platform, acquiring all data between January 2016 and February 2019. We then prefiltered the data set to remove content deleted by either moderators or users, as indicated by the *[deleted]* and *[removed]* tags. We also removed content posted by the subreddit’s moderators and the user u/AutoModerator, a Reddit bot designed to automate moderation tasks.

Next, we selected 1000 posts to build an annotation data set, randomly sampled without replacement, for constructing all piloting data sets and the final annotation data set. We discarded posts that had short (>5 words, including the title) or long text content (>1500 words), as requested after a few rounds of piloting by 2 members of the research team. Short posts were removed because of the difficulty in providing meaningful annotations about the risk or protective factors; annotators found it difficult to evaluate concrete factors with no details. Long posts often contained so much information that they overwhelmed the labeling schema. Together, these posts were very rare in our sample of 1000 posts—<20 posts or <2%. We also manually inspected each post to remove those that asked for help on behalf of someone else or that were about suicide bereavement (around 10 posts or 1%). We then gathered descriptive statistics for our data set, as shown in Table 1.

**Table 1:** Summary statistics for the 1000-candidate post data set.

Characteristics	Values
Total number of unique users	984
Post length (words), mean (SD)	221.81 (267.98)
Median length	141

#### Data Deidentification

Following best practices for detailed annotation of suicide content [15,33,39], we deidentified each post to remove personal details. We first removed any mention of usernames or links from the posts. Next, we tagged all person, organization, or location names using Stanford’s Named Entity Recognizer through the nltk Python library. We replaced any tagged words with placeholder text (eg, named locations with the term LOCATION). To verify that these data were deidentified, the researcher responsible for gathering the data set manually checked and edited any posts to remove identifiable information, as necessary. After this step, the data were passed to the broader research team for coding.

#### Research Team and Positionality

The research team included 4 experts with complementary experiences across social media, mental health, public health, and suicide. This approach represents an interdisciplinary collaboration that considers public health, clinical, and social computing perspectives.

A total of 2 researchers are public health experts with additional backgrounds in psychology and clinical medicine. The other 2 researchers are computer scientists who are experts in social media and mental health. The team also included people with lived experiences of mental illness. Together, they have extensive experience working in high-risk mental health behaviors, such as suicide, expressed through social media.

#### Designing the Annotation Scheme

##### Phase 1: Evaluation of Context and Preliminary Item Development

To begin the initial development, we drew on several sources to understand the risk and protective factors. First, we reviewed the classification schemes used by the Centers for Disease Control and Prevention’s National Violent Death Reporting System (NVDRS). NVDRS is a state-based system that collects data from multiple sources (eg, death certificates, coroner and medical examiner reports, law enforcement, and toxicology reports) to provide context for violence-related deaths, including suicide [40]. The NVDRS collects information about many risk factors for suicide, such as preceding health and mental health problems, as well as social and environmental factors associated with suicide. In addition, the research team drew on other work in suicide and social media [13,41], the relevant literature on risk and protective factors [40,42], and their experiences engaging with online mental health communities to create an initial version of the scheme.

This annotation scheme included questions related to suicidality and public health. Each item contained an overview defining and clarifying the item and excluding other categories. For example, the risk factor “crisis in past 2 weeks or upcoming 2 weeks” was paired with the following text for annotators: “Direct language that the event caused or contributed to the suicidal ideation or behavior is not required to code ‘yes’. Use judgment to determine the time frame. Variable may overlap with other categories (eg, house foreclosure, court date for criminal offense).”

In addition to risk and protective factors, we also captured supplementary information useful in contextualizing risk and protective factors and complementing the use of this survey by stakeholders in suicide prevention (eg, national public health authorities, web-based moderators, and supportive others). The literature points to discussion or intentions with methods of harm as a key part of assessing intention and risk; therefore, we developed an item related to potential methods that an individual may discuss. We also included demographic information volunteered by the poster in r/SW, including self-stated gender and age, as well as whether the poster states that they are in the United States. See Multimedia Appendix 1 for the extended items, definitions, and clarifications of the annotators.

##### Initial Piloting and Adjustments

A total of 2 members of the team then independently piloted the scheme on 25 random posts drawn from the candidate data set. A total of 2 undergraduate research assistants also piloted the scheme for clarity and interpretability. The 2 members of the research team reported taking 30-45 minutes on the task, and the undergraduates took longer, between 45 minutes and an hour. All took detailed notes on their experiences; then, the team discussed their findings to come to a consensus and refined the scheme based on content:

- Assumptions around depressed mood: there was substantial conversation around annotating if the poster had “depressed mood and mental health problems.” The nature of posting in a suicide crisis forum would be inferred to indicate the presence of suicidal thoughts or considerations and some common mental health conditions, such as depression. On the basis of the pilot and to increase precision and sensitivity to identifying mental health conditions that may be contributing to suicide risk, the initial risk factor of “depressed mood and mental health” was refocused to include only explicit mentions of mental health diagnosis and symptoms other than suicidality.
- Write-in risk and protective factors: the annotation task presented situations where the annotators found risk or protective factors unaccounted for in the categories, though not prevalent enough to warrant a separate category (such as care for family members and dependents). For these, we added a write-in option to the risk and protective factor questions.

During this iteration phase, we also refined the scheme for practical concerns with labeling:

- *Unable to determine* signifier: for demographic questions, we added an option of *cannot tell/not indicated* to assist annotators in indicating their confidence that not enough information was provided to assess the poster’s gender, age category, or possible location.
- Removed *any risk or protective factor present* category: this category was duplicated with other labels in the other categories, and the annotators did not feel it was useful to potential future efforts to connect factors to suicide interventions.

##### Phase 2: Formal Testing and Refinement, Initial Evaluations

After the initial version of the scheme was piloted, 2 team members annotated three rounds of posts randomly sampled without replacement from our candidate data set. Each time, they annotated 20-25 posts and then began checking for internal agreement. Between each round, all researchers met to clarify inconsistencies and better separate categories. Inconsistencies often involved discussing a single post's annotations or how to finesse the descriptions and definitions of items to strengthen consensus.

##### Interrater Agreement and Reliability Measurements

To evaluate internal validity, we selected 20 posts that were independently annotated by 2 raters on the team with experience in public health and mental health. For categories that were borderline on good-to-strong agreement, we supplemented those with an additional 20 posts for annotation. To quantitatively evaluate the agreement for each subitem or question, we used Gwet AC-1 over Cohen κ or raw percentage agreement. Although Cohen κ is frequently used for interrater reliability evaluations [43], Cohen κ does not adjust for rare category representations within data sets [44,45]. Gwet AC-1 manages rare or infrequent events better than Cohen κ and avoids the pitfalls of large class sizes when evaluating straightforward percentage agreements. In the final version, we saw strong agreement (Gwet AC-1>0.6) across all but one item (*explicit statement of mental health symptoms or diagnosis other than suicidality*).

#### Final Ratings for 200 Posts and Exploratory Factor Analysis

After establishing interrater agreement and consistency for evaluation, the 2 annotators rated 100 posts each. They rated these items independently, and we counted their annotations together for a total of 200 posts. The expert raters reported that this took between 1 and 3 minutes per post, depending on the post's length. The results from this analysis are presented in Table 2.

**Table 2:** Independent annotations of 200 posts (N=200).

Annotation category		Values, n (%)
**Risk factors (included at least one)**		164 (82)
	Crisis in past 2 weeks or upcoming 2 weeks	11 (5.5)
	Social or relationship problem	102 (51)
	Finance or job problem	48 (24)
	Physical health problem	18 (9)
	Alcohol dependence	10 (5)
	Other substance use problem	11 (5.5)
	Legal problem	4 (2)
	School- or academic-related problem	26 (13)
	Death of a friend or family member	7 (3.5)
	Explicit statement of mental health symptoms or diagnosis other than suicidality	98 (49)
	History of abuse or witnessing violence in childhood	17 (8.5)
**Protective factors (included at least one)**		128 (64)
	Positive social support presence in life	92 (46)
	Desire to get better or feel better	59 (29.5)
	Lack of means to harm self (perceived or actual)	8 (4)
	Engagement in activities	15 (7.5)
	Sense of purpose or hope	9 (4.5)
	Access to health or mental health care	33 (16.5)
**Gender**		86 (43)
	Male	48 (24)
	Female	29 (14.5)
	Transgender	9 (4.5)
	Cannot tell or not indicated	114 (57)
**Age (years)**		101 (50.5)
	High school or younger (<18)	24 (12)
	College (18-22)	21 (10.5)
	Postcollege (23-29)	14 (7)
	Young adult unspecified (any age<30)	30 (15)
	Adult (>30)	12 (6)
	Cannot tell or not indicated	99 (49.5)
**Mechanism**		62 (31)
	Firearm	12 (6)
	Suffocation, hanging, or strangulation	11 (5.5)
	Poisoning	23 (11.5)
	Harm using sharp instruments or cutting	23 (11.5)
	Fire or burns	0 (0)
	Fall	9 (4.5)
	Drowning	3 (1.5)
	Motor vehicle or train accident	7 (3.5)
**Post from inside the United States**		189 (94.5)
	Yes	175 (87.5)
	No	14 (7)
	Cannot tell or not indicated	11 (5.5)

Finally, we conducted a correlational analysis using tetrachoric correlations and exploratory factor analysis (EFA) to determine the relationships between individual protective and risk factors. EFA is a technique commonly used in scale development and psychometrics to evaluate whether any variables in a scheme, survey, or instrument are correlated such that they may be explained by unobservable or underlying variables called *factors* [46]*.* This allows us to inspect for potential overlap with correlations and how the schema may be reduced in future work. For EFA, we separated risk and protective factors, as these items were developed distinct from each other and had the most potential for common concepts and underlying factors. We conducted EFA using minimum residuals on the tetrachoric correlational matrix, and we reported the results using parallel analysis [46]. These are available in the psych package in R (R Core Team).

## Results

### Agreement Between 2 Raters

The final scheme items and their agreement scores are listed in Table 3. We saw strong results that indicated our annotation scheme was consistent between the 2 raters.

**Table 3:** Interrater reliability between raters in the final data set.

Question		Agreement, %	AC-1
**Risk factors**			
	Crisis in past 2 weeks or upcoming 2 weeks	90	0.87
	Social or relationship problem	81	0.64
	Finance or job problem	90	0.85
	Physical health problem	95	0.94
	Alcohol dependence	100	1
	Other substance use problem	98	0.97
	Legal problem	100	1
	School- or academic-related problem	90	0.86
	Death of a friend or family member	100	1
	Explicit statement of mental health symptoms or diagnosis other than suicidality^a^	78	0.57
	History of abuse or witnessing violence in childhood	100	1
**Protective factors**			
	Positive social support present in life^a^	90	0.81
	Desire to get better or feel better^a^	80	0.68
	Lack of means to harm self (perceived or actual)	95	0.95
	Engagement in activities	93	0.92
	Sense of purpose or hope	88	0.86
	Access to physical or mental health care	90	0.86
**Gender**			
	Male	93	0.89
	Female	100	1
	Transgender	100	1
	Cannot tell or not indicated	90	0.81
**Age (years)**			
	High school or younger (<18)	98	0.97
	College (18-22)	98	0.97
	Postcollege (23-29)	98	0.97
	Young adult unspecified (any age<30)	88	0.83
	Adult (>30)	100	1
	Cannot tell or not indicated	85	0.72
**Method**			
	Firearm	98	0.97
	Suffocation, hanging, or strangulation	nan^b^	nan
	Poisoning	98	0.97
	Sharp instrument or cutting	100	1
	Fire or burns	nan	nan
	Fall	98	0.97
	Drowning	100	1
	Motor vehicle or train	nan	nan
**Post in the United States^a^**			
	Yes	80	0.71
	No	95	0.94
	Cannot tell or not indicated	85	0.81

^a^Indicates where we added 20 posts in the final rating for disambiguating challenging categories.

^b^Indicates that it was not present in the annotations.

### Results of Annotation for 200 Posts

In Table 2, we present the independent annotations of 200 posts. We provide quotes for the context that has been edited to protect participants’ identities.

Out of 200 posts, about 164 (82%) posts included at least one risk factor, and 128 (64%) posts included at least one protective factor. Related to this, 31% (62/200) described a possible mechanism for a current or past attempt at suicide. Of all 200 posts, 189 (94.5%) had either a protective or risk factor, leaving only 11 (5.5%) posts that did not. We manually inspected these 11 posts, and these tended to be very short posts with little information about the person’s unique circumstances (eg, the entire post was “that calm when you finally decided–yea i’m gonna do it”). We noted that very short posts were difficult for annotators because of their length, and this limited the ability to apply this scheme to them. However, most posts were rich enough for annotation by the data set and indicated that community members were willing to disclose suicide risk and protective factors.

### Analysis of Risk and Protective Factors

In Tables 4 and 5, we present histograms of the count of risk and protective factors by post. This shows that many posters have more than one risk or protective factor that they mention, indicating multiple avenues for support that may not have been captured through the evaluation of risk in a binary classification system.

**Table 4:** Risk factors per post.

Number of risk factors present in post	Count in data set
0	36
1	56
2	49
3	35
4	14
5	8
6	2
7	0

**Table 5:** Protective factors per post.

Number of protective factors present in post	Count in data set
0	72
1	65
2	41
3	16
4	6
5	0

The most prevalent risk factors were social or relationship problems (102/200, 51%), mental health symptoms (98/200, 49%), financial or job problems (48/200, 24%), school or academic problems (26/200, 13%), and physical health problems (18/200, 9%). We noted that over half of all posts mentioned social and relationship problems in their posts. These included trouble with family members (“I can’t stay with my family for another 10 months”)*,* breakups (“I miss my ex so much, but he doesn’t care about me and has forgotten me with his new girlfriend”), and the absence of relationships and friends (“I can’t really say I’ve had a friend in the last 5 years”). Combinations of these factors also included navigating the devastating effects that mental health symptoms have on relationships and friendships (“I’ve tried to hide my depression from my friends for years, but my best friend is so exhausted dealing with me. She must know by this point...”). For mental health symptoms, many noted that their symptoms recurred or were not well treated or that their relationship with their therapist or medical professional was not supportive. Write-in examples for risk factors were varied and included circumstances such as cutting and self-injury and losing access to technology.

The most prevalent protective factors were positive social support (92/200, 46%), desire to get or feel better (59/200, 29.5%), access to health or mental health care (33/200, 16.5%), and engagement in activities (15/200, 7.5%). Examples of posts that indicated positive social support included support from family and friends (“A friend recommended me for a job”) and worries about disappointing supportive people in their lives (“I’m so scared because my mom couldn’t handle it if I weren’t here, but I’m so miserable.”*).* Those who desired to get or feel better described both the active desire to get better (“I want to believe that I’ll get through these feelings”) and the negative desire to not die (“I really don’t want to die, but I can’t keep living like this either”). In addition to these categories, our annotators also identified other protective factors mentioned by the posters, such as how their religion discouraged suicide as a solution.

### Analysis of Demographic and Methods Factors

Only 43% (86/200) of posts included a discernable indication of their gender. Of the posters who mentioned their gender, 56% (48/86) were male. We noticed that gender was mentioned in the post body (eg, “hi I’m 26/M and struggling”), in the context of risk or protective factors (“there’s no way I’m better than the other men she loves”), or in the mental health struggles they were currently encountering (“my gender dysphoria is very bad tonight, please help”). Of the 200 posts, 9 (4.5%) had people who identified as transgender, gender fluid, or nonbinary identities. These individuals often described being closeted for their true gender or frustrations around being misgendered.

As for age, approximately half of the posts (101/200, 50.5%) indicated the person’s age group. The largest age group on the forum was young adults aged >30 years, with many of them being in college (21/200, 10.5%), and some being in high school (24/200, 12%). Many posts mentioned age in passing, with no connection to circumstances surrounding their ideation (“19yo—please help”). However, some posts often related to age as a factor for both risk *(*“I’m 58 and I’ve wasted my whole life”) and protective (“I know I’ll graduate [college] soon, and then it’ll be easier”) factors.

About 31% (62/200) of total posts described a possible mechanism for a current or past attempt at suicide. For posts that include a mechanism, these posts mention only one, and those in descending order are poisoning (23/200, 11.5%), sharp instruments and cutting (23/200, 11.5%), and self-injury via firearms (12/200, 6%). Some of these posts mention it in the context of past attempts (“I tried to drown myself”) or in present or future possibilities.

Finally, we examined whether posts indicated that they were in the United States. We found that 87.5% (175/200) of posts were inferred to be from posters in the United States. As we removed location details from the posts, this category was generated mostly from inferences about context. This included current details (“I make about 30k a year”) or past history of the participant (“we moved a lot between k-12”). Other contextual details were indicative for people not in the United States, such as the context of learning a new language after moving to a new and small country, or other personal indicators (“I only weigh 8 stone”).

### Item Correlations and Factor Analysis on 200 Posts’ Ratings

**Figure 2:**
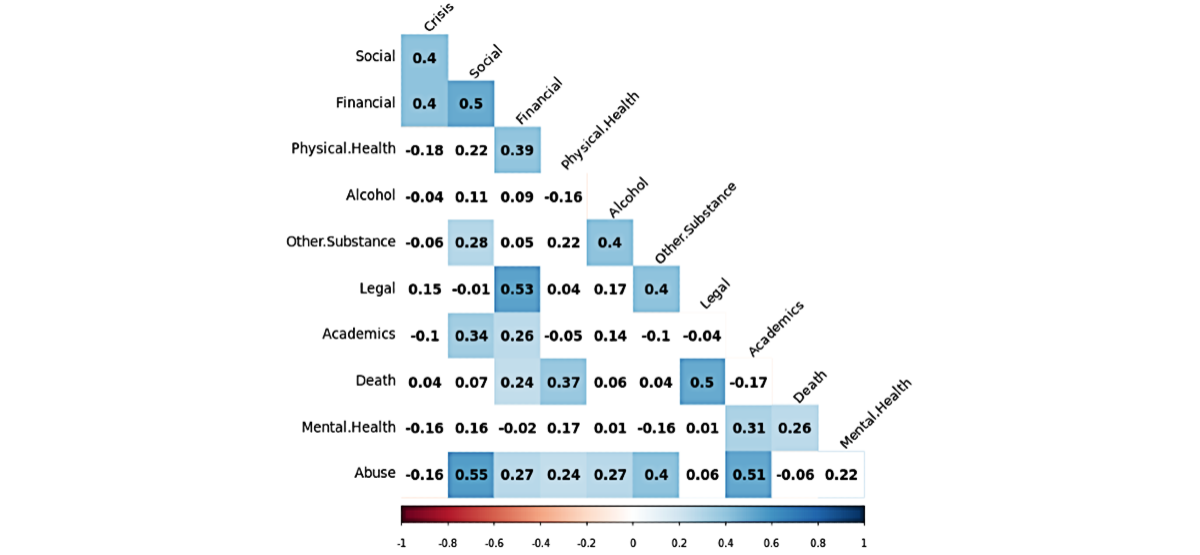
Tetrachoric correlations between risk factors.

**Figure 3:**
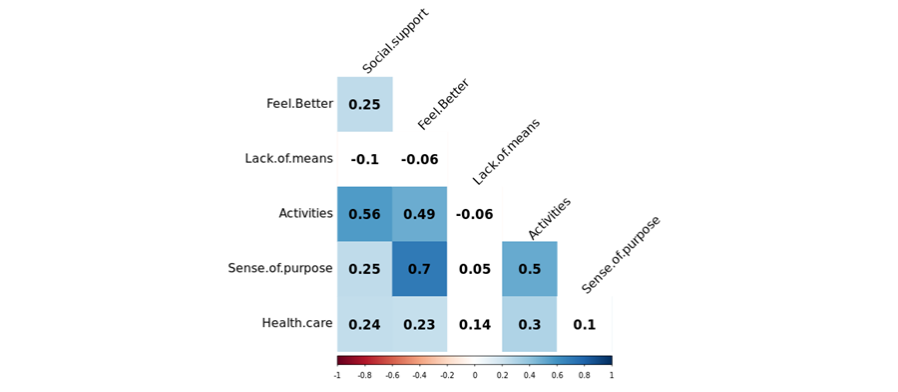
Tetrachoric correlations between protective factors.

First, we present the correlations of the variables in Figures 2 and 3 using tetrachoric correlations. Tetrachoric correlations are useful for measuring the strength of correlations between binary or dichotomous data. Colored or shaded cells indicate significance at the *P*<.01 level after applying the Benjamini-Yekutieli correction to account for false discovery rate.

Many variables show correlations with other risk and protective factors. Some correlations are relatively strong, such as the correlation between legal and financial concerns (*r*=0.53) and abuse and social factors (*r*=0.54). The correlations of the factors themselves are not surprising based on prior work on suicide prevention, as many factors are independently related to each other [47]. For instance, research has shown the impact of abuse and violence in childhood, commonly reframed from adverse childhood experiences, and their connections to negative outcomes in adulthood [48], such as alcohol (*r*=0.27) and substance abuse (*r*=0.4). We saw similar correlational strengths in the protective factors. There is a very strong correlation between the complementary factors of having a sense of purpose in one’s life and the ability to feel better (*r*=0.70). We noted the distinctiveness of the lack of means to harm oneself, which showed no significant correlations with any other factors. We hypothesized that this factor might be distinctive from the others, and future work should explore the independence of this factor.

**Figure 4:**
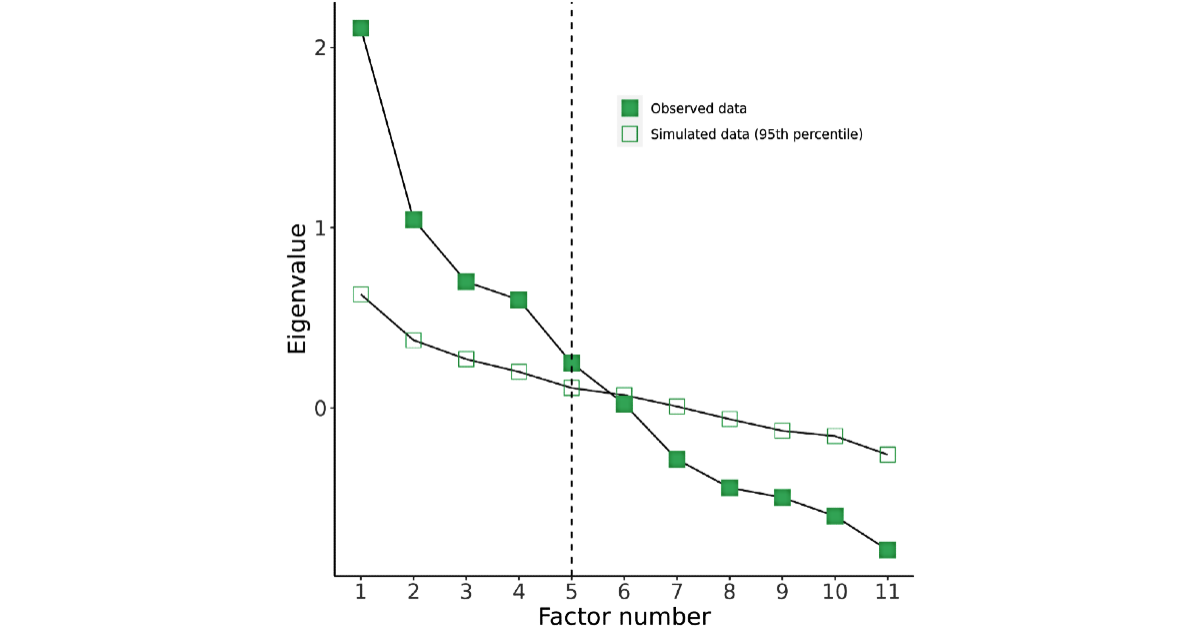
Scree plot and parallel analysis for risk factors.

**Figure 5:**
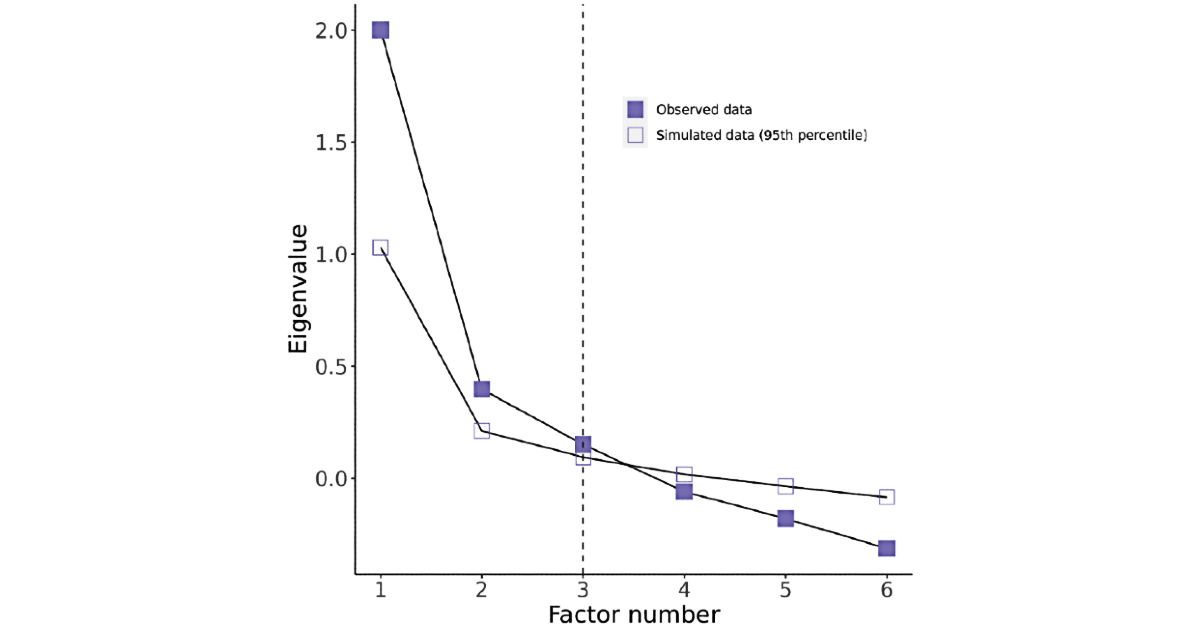
Scree plot and parallel analysis for protective factors.

Next, we present the EFA for our analysis, relying on the tetrachoric correlations we used earlier. We opted for parallel analysis rather than the scree plot of the eigenvalues as there was no distinctive *elbow* for risk factors, a common signal for effective interpretations of scree plots, as shown in Figures 4 and 5 for risk and protective factors, respectively. Parallel analysis is a complementary evaluation technique to scree plots that use simulated data to evaluate factor reduction [46]. Parallel analysis pointed to five unobserved factors for risk and 3 unobserved factors for protection. Both models have a reasonable percentage of variance explained—73% of variance explained for a reduced model of five risk factors and 57% of variance explained for a reduced model of three protective factors (we expect between 60% and 70% of variance explained, per DeVellis [46]). This aligns with our conceptual model that there is distinctiveness among the socioecological factors proposed in prior work.

## Discussion

### Principal Findings

Our work responds to recent calls within computer science to better operationalize concepts for social media analysis [25-27]. We did so through the synthesis of procedures, vocabulary, and perspectives of measurement and validity literature in linguistics [28] and psychometrics [30,31]. We have provided a more formalized, step-by-step approach to generate annotations for social media analysis for mental health.

This work affects public health and computational research by better conceptualizing risk and protective factors that influence suicidality. Leading public health data about suicidality are largely generated from suicide decedents, and information on precise circumstances and precipitants influencing individuals at risk of suicide is lacking. Large-scale information on suicide ideation is valuable as it provides information on a time point that is upstream of significant morbidity. This may help improve public health programs to prevent suicide, such as programs to enhance protective factors such as social connectedness [49].

Furthermore, this research points to improvements in the computational analysis of social media data for mental health and suicide. With more testing and a larger volume of samples, we envision that our annotation scheme can be used in semiautomated machine learning systems that screen natural language for mention of risk and protective factors. These systems could eventually direct support and resources to those with more urgent risk or with complementary protective factors that may assist in mitigating crisis (such as shared experiences and social connections) [1].

### Comparison With Previous Work

Social media data have been a fruitful data source for research on suicide. Many studies have attempted to distinguish whether an individual is suicidal [14,41,50] or may attempt suicide in the future [51]. Early research by Coppersmith et al [51] used the disclosure of a past suicide attempt to understand the pathology of risk. Another study identified 6 categories of suicide-related disclosures, such as public awareness campaigns and memorial campaigns alongside legitimate disclosures [41]. De Choudhury et al [5] studied shifts in Reddit to suicidal ideation from other mental health subreddits, and Kavuluru et al [38] designed a classification approach to detect helpful comments on r/SuicideWatch [37].

The dominant model for understanding suicide on social media has focused on risk, operationalized into categories of low, medium, or high. In early work, Homan et al [14] adopted a manual annotation process to verify the veracity and trustworthiness for clinical diagnosis of a mental disorder that is then fed to language models to understand distress and suicide risk. These annotation surveys and schemes are in service of annotation for automated or artificial intelligence systems. O’Dea et al [12] used Twitter data to develop an annotation scheme for mixed-expertise coders to annotate among *strongly concerning, possibly concerning,* or *safe to ignore*, which is then used for prediction. Building off shared tasks in natural language processing [52,53], both Milne et al [54] and Cohan et al [7] designed a survey that annotated with the *stoplight* system of green-amber-red-crisis, which was then fed to machine learning models to improve moderator responsiveness on ReachOut. Closest to our study, Shing et al [13] developed an ordinal risk assessment annotation with four categories for Reddit suicide crisis data, with mental health experts as annotators compared with the crowd.

An active area of research innovates in strategies for assessing mental health signals and generating labels. Some studies used trained medical professionals to generate labels [12,13]. Although this approach is promising because of its direct connection to everyday clinical practice, scaling this specialized skill to the number of posts needed for stable social media analysis is burdensome for clinicians with outside responsibilities and busy schedules [24]. Researchers have developed scalable labeling strategies for use by people other than clinicians, which have been called *proxy signals* in prior work [24,33]. Although these approaches aim to distribute labor, recent work has called into question whether these proxy signals measure what they claim to measure. Ernala et al [24] empirically compared the outcomes of proxy signals derived from prior work and found that computational models had poor external validity on verified patient data for patients with schizophrenia. Similarly, Chancellor and De Choudhury [23] found that there has been little research evaluating clinical constructs in social media data.

Together, this research points to gaps in the approaches to the annotation of suicidal behaviors. As social media data do not, by default, include clinically validated labels of suicide, processes relying on these signals must be replicable and reliable. Our study responds to and makes the first attempt at reconciling these criticisms in a labeling task designed to annotate risk and protective factors in social media data.

### Considerations for Developing New Schema for Annotation

We believe our approach can be extended to other cases where teams need high-quality annotations from social media data in mental health and beyond. In this section, we provide an overview of the considerations and guiding questions to adopt this framework in new schema development.

#### Problem Framing and Domain Expertise

Problem framing is both the origin and evaluation point for research and practice and is a core component of construct validity. Although computer scientists are experts in technical methods and social media, they do not carry the same background, intuition, and framing expertise as psychiatrists and psychologists, researchers in medicine and psychology, social workers, or other experts. The right set of domain experts can make it clear how to instantiate certain concepts in surveys and adjust and evaluate concepts to align with notions of construct validity. Do our definitions of illness or behavior hold up to appropriate disciplinary scrutiny [23]? What, specifically, is the exact problem to evaluate? We strongly encourage working with experts in mental health as a de facto standard in work that bridges mental illness and computer science to assist with questions of construct validity.

#### Source of Social Media Data

In addition to problem framing, the social media data source will need to be evaluated for its capacity to provide insight into a question. Different platforms and affordances, subcommunities, and normative practices may lend themselves to answering certain kinds of questions about mental health and human behavior. Can social media data from a specific platform answer the question that the team wants to solve, or do modifications need to be made to the community data source, platform, or questions being asked?

#### Automated and Deliberate Filtering

Social media data are almost always processed, filtered, or curated by both the platform and the research team. Data gathering techniques may be altered by the platform, preventing the curation of a truly random sample (Twitter data streams typically provide 1%-5% of all data), and research teams may choose to remove posts that do not meet certain objective criteria for length or language patterns. For instance, we chose to remove very short and very long posts from our annotations from expert requests. What are the impacts of different kinds of filtering on the generalizability of the findings or schema?

#### The Tradeoff Between Complexity and Validity

A crucial balancing act will come between the complexity of the schema and the schema’s validity. We anticipate that a schema that has the highest levels of construct validity will also be time consuming given the schema’s length. Simplification may be necessary to reduce time and resource costs. The same simplification can quickly become a dangerous abstraction that loses the ability to evaluate the original concept, a shadow of its original concepts, and may lead to erroneous conclusions. However, the simplification of a schema to core factors is a subfield of psychology. Methods such as EFA can assist in the process of robustly condensing schema, but this requires the appropriate use of new methods. What are the most robust strategies for managing complexity and validity?

#### Who Does the Labeling?

In addition to the development of schema, the actual people and groups that label social media data are just as important as the development of the schema itself. Domain experts are ideally the best to label posts; however, their time is valuable and constrained. In computer science, researchers have turned to nondomain experts and crowd workers on sites such as Mechanical Turk to quickly label large batches of data [13].

Although crowd workers have been adopted for tasks such as image labeling, their utility in subjective tasks such as mental health evaluations is a nascent area of study. Most studies use simplified versions of risk evaluations, such as the promising work of Shing et al [13] in suicide risk assessment, and do not use an expanded schema similar to ours. Who are the best sources of labels for robust schema, and how do they diverge from more accurate assessments? What thresholds of accuracy from different groups are appropriate for evaluating social media data?

#### Resource Management

In ideal production scenarios, there would be plenty of time to develop a robust schema and test and label thousands of posts. However, this is infeasible in practice, especially in professional environments where the time of experts is limited, and costs may drive decision-making. What resource tradeoffs are appropriate for maintaining quality standards? How many high-quality annotations can be generated, and does this ensure that a model or finding is robust? What emerging ethical and moral questions arise from the tradeoffs required for resource management?

#### Software for Annotation Schemas

There are many ways to format and deploy a schema through software that may have secondary impacts on time to completion, perceived complexity, and accuracy. Work in the field of human-computer interaction and crowd working considers how technical design tradeoffs may affect these variables. For example, our raters found Google Forms burdensome in prepiloting; hence, we abandoned it. We encourage mindfulness of these methods and concerns to avoid unforeseen interactions between tools and technology with the annotation schema itself.

### Ethics and Privacy Considerations

We believe that all researchers have obligations to protect individuals in their data sets from harm, no matter the source of the data or protections or exemptions from ethics boards [33]. We followed emerging professional and research norms of care for social media data in sensitive contexts [33,39]. However, careful research protections do not inherently guarantee that the participants will not be reidentified, that the research process is human-centered, or that the implications will generate just outcomes for individuals whose data are analyzed [36]. Tensions in scientific reproducibility, moral imperatives for intervention, professional ethics obligations, and other factors emerge when dealing with challenging areas such as suicide prevention [15,33].

One tension in development is the balance between the inclusion of correct gender identities and risks of harm from a small sample size. Understanding a person’s gender identity is an important facet of suicide prevention, as suicidal ideation disproportionately affects LGBTQ+ individuals [55]. We considered including more inclusive gender categories recommended by experts [56], including nonbinary and genderfluid identities. On manual inspection of the data set, there were very few individuals who self-described as nonbinary or genderfluid—<2% of all posts (2-3 posts of 200). We worried that our research could have a spotlighting effect on their behavior if we chose to isolate these gender categories and present comparisons of these individuals because of the small data set size. This attention risks harming individuals who may already be vulnerable for reasons related to their gender identity and poor social support from others. Therefore, we opted to bundle the identity categories together and label individuals who were genderqueer, genderfluid, and transgender as one category.

### Limitations and Future Work

The primary limitation of this approach is that external validation of this annotation scheme is needed. This includes robust confirmatory factor analysis, reduction of factors into more generalized concepts, and a scale evaluation and deployment with new raters and a new data set. This facet of generalizability is important for benchmarking the performance of downstream applications. Our study focuses on the critical first step of establishing construct validity in annotation development, and our immediate and future work will focus on demonstrating external validity with new annotators and communities discussing suicide crises.

Other data concerns that might limit generalizability are connected to this concern. Although it is anchored in broader concepts for suicide risk and protective factors, this scheme was developed for the unique context of Reddit suicide crisis posts. Reddit demographics will skew toward younger audiences, may be biased for US contexts, and may miss crucial demographics at risk for suicide. We also expect explicit requests for assistance through a suicide crisis to influence our annotation schema and not translate as well to more subtle disclosures. We do not yet have a sufficient sample size to present a generalizable analysis of differences in risk and protective factors differing between different demographic factors or across cultural differences in expressions of mental illness. Future work will additionally need to extend and verify this scheme on new communities and platforms such as mental illness subreddits more broadly (eg, r/depression, r/selfharm, and r/madeofstyrofoam) or general-purpose social media sites.

However, these limitations are opportunities for future research. As a crucial next step, external validity should be added to this pipeline to develop our approach. New individuals could be involved as raters, including novices and computer scientists with domain expertise but no medical training, crowd workers, and other experts and stakeholders in this space. This would complement previous studies by comparing experts and nonexperts [13,14]. In addition, we could consider validating these data against other sources of information about suicide, such as public health data sets or alternate social media sites and communities.

### Conclusions

We report the development and first validation of an annotation scheme that evaluates risk and protective factors in suicide crisis forums on Reddit. By using the socioecological model of suicide prevention, our approach expanded state-of-the-art processes by moving beyond categorical or ordinal scales to evaluate only risk. Moreover, by adding protective factors to the scheme, we provided key insights into behavior that better represents the constellation of support needs for suicide prevention. Aligning with the metrics of construct validity, we demonstrated strong substantive and structural agreement among the research team. By explicating our processes, logic, and decision-making, we not only hope to enable replicability in social media annotation of suicide but also raise awareness for annotation development.

## Appendix

Multimedia Appendix 1 Extended details of the annotation schema developed for this study.

## Abbreviations

EFA: exploratory factor analysis
NVDRS: National Violent Death Reporting System
r/SW: r/SuicideWatch
