# Supplementary material for: Suicide Risk and Protective Factors in Online Support Forum Posts: Annotation Scheme Development and Validation Study
Source: JMIR Ment Health. 2021 Nov 8;8(11):e24471. doi: 10.2196/24471 (PMC8663675; doi:10.2196/24471)
Supplement: Multimedia Appendix 1 [file mental_v8i11e24471_app1.docx]

## Appendix

We include the complete item definition and details here.

| **Precipitating Circumstances/Triggers** | |
| --- | --- |
| **Category and Items** | **Definition** |
| Crisis in past 2 weeks or upcoming 2 weeks | Direct language that the event caused or contributed to the suicidal ideation or behavior is not required to code “Yes”. Use judgement to determine time frame. Variable may overlap with other categories (e.g., house foreclosure, court date for criminal offense). |
| Social/Relationship Problem | Problems with a current or former intimate partner, family member, friend/associate, peers appear to have contributed to suicidal ideation or behavior. Includes problems making or maintaining peer relationships and general descriptions of feeling socially inept or awkward. |
| Finance/job problem | A financial problem appears to have contributed to suicidal ideation or behavior. Financial problems may include a recent eviction or other loss of housing, or the threat of it; experiencing a problem at work (such as tensions with a co-worker, poor performance reviews, increased pressure, feared layoff) or was having a problem with joblessness (e.g., recently laid off, having difficulty finding a job); or other financial problem (recent loss of business, bankruptcy). |
| Physical health problem | Chronic and acute health conditions. Do not include mental health or substance use problems; pain or chronic pain would be considered a physical health problem |
| Alcohol Dependence | Perceived by self or others to have a problem with, or to be addicted to, alcohol; must be a current problem |
| Other Substance Problem | Perceived by self or others to have a problem with, or to be addicted to drugs other than alcohol; must be a current problem and can include illegal, prescription, or over the counter substances |
| Legal problem | Criminal or civil legal problem appear to have contributed to the suicidal ideation or behavior. Criminal legal problems are those resulting from conduct considered as harmful to society that it is prohibited by statute and prosecuted by the government (e.g., DUI, robbery). Committing a crime alone is not sufficient basis for endorsing this variable; there must be evidence of negative legal or law enforcement consequences (e.g., about to enter jail, facing a court date, on the run from law enforcement) that appear to be associated with the suicidal ideation or behavior. Examples of civil legal (non-criminal) problems include the victim being upset because they lost custody of their child the day before the suicide attempt or the victim losing a civil lawsuit the week before suicide attempt. |
| School or academic-related problem | Problems at or related to school. Examples include poor grades, difficulty with a teacher, bullying, social exclusion at school, school detention/suspension, or performance pressures |
| Death of friend or family member | Death of a family member or friend due to any cause, including suicide. Any time frame applicable if event contributed to current state |
| Explicit statement of mental health symptoms or diagnosis other than suicidality | Individual perceived by self or others to be depressed or to have a mental health disorder or syndrome, such as anxiety disorder, bipolar, depressive disorder, schizophrenia, eating disorder, or obsessive-compulsive disorder. Does not need to be a formal diagnosis and may include symptoms of being sad, despondent, down, blue, low, unhappy, loneliness, hopelessnesss, sleep problems, etc. Do not include alcohol or substance dependence here. Do not include explicit statements of suicidality, such as wanting to die or kill oneself. |
| History of childhood abuse (sexual, physical, psychological, or neglect) or witnessing violence in childhood (before age 18) | Definitions of various forms of childhood abuse include one or more of the following definitions in italics: |
| *Sexual abuse* | Any completed or attempted (non-completed) sexual act, sexual contact with, or exploitation (i.e., noncontact sexual interaction) of a child by a caregiver, family member, or relative |
| *Physical Abuse* | The intentional use of physical force against a child that results in, or has the potential to result in, physical injury. Physical abuse can result from discipline or physical punishment and can include hitting, kicking, punching, beating, stabbing, biting, pushing, shoving, throwing, pulling, dragging, dropping, shaking, strangling/choking, smothering, burning, scalding, and poisoning by a caregiver, family member, or relative |
| *Psychological abuse* | Intentional caregiver behavior that conveys to a child that he/she is worthless, flawed, unloved, unwanted, endangered, or valued only in meeting another’s need. Examples include blaming, belittling, degrading, intimidating, terrorizing, isolating, restraining, confining, corrupting, exploiting, spurning, or otherwise behaving in a manner that is harmful, potentially harmful, or insensitive to the child’s developmental needs, or can potentially damage the child psychologically or emotionally by a caregiver, family member, or relative |
| *Neglect* | Failure by a caregiver to meet a child’s basic physical (e.g., nutrition, hygiene, shelter), emotional (e.g., ignores, not emotionally responsive to child), medical/dental, or educational needs. Neglect also includes the failure by the caregiver to ensure a child’s safety within and outside the home given the child’s emotional and developmental needs (e.g., inadequate supervision, exposure to violent environments) |
| *Witnessing Violence* | Witnessing physical violence at home (such as father beating mother), in the community (such as gang or street violence), at school (such as frequent physical fights), or other locations |
| Other circumstance/triggers (write in) |  |
| **Protective Factors** | |
| Positive social support present in life (family, spouse/intimate partners, friend, peer, community member) | Mark if support person is present, available, or trying to help. Include if individual reports social support or not wanting to hurt those providing social support is a reason for not completing suicide. Do not code as if support person is unavailable or perceived to be harming individual. |
| Desire to get better/feel better | The individual indicates that they want to feel or get better. This includes indicating they want to feel better, looking for other groups or individuals for support. This also includes pleas/desire for someone to talk them out of harming themselves. |
| Lack of means to harm self (perceived or actual) | Individual does not feel they have access to desired means for suicide (e.g., no access to pills) |
| Engagement in activities | Activities engaged in by person beyond work or school. May include clubs, volunteering, civic or religious group participation. Also include active participation in physical activities which are a source of support to individual, such as sports (inside or outside of school), martial arts, weightlifting, hiking, or any physical exercise. |
| Sense of purpose or hope | Indications of hopefulness, optimism, and having a life purpose. Can include any future goals for employment, relationships, and achievements. |
| Access to health/mental health care | Mentions current treatment by a health or mental health professional to address symptoms or circumstances contributing to the thoughts of suicide. Such professionals may include therapist, counselor, psychologist, doctor, or other clinician. Mark this even if the person feels that treatment is not working. |
| Other circumstance/triggers (**write in**) |  |
| **Gender** | |
| Male |  |
| Female |  |
| Transgender |  |
| Cannot tell/not indicated |  |
| **Estimated Age Group** | |
| High school or younger (<18) |  |
| College (18-22) |  |
| Post-College (23-29) |  |
| Young adult unspecified (any age <30) |  |
| Adult (30+) |  |
| Cannot tell/not indicated |  |
| **Mechanism of contemplated/planned (or previous) suicide attempt** | |
| Firearm | Method that uses a powder charge to fire a projectile |
| Suffocation/Hanging/Strangulation | Such as hanging by the neck, manual strangulation, or plastic bag over the head |
| Poisoning | Includes street drug, alcohol, pharmaceutical, carbon monoxide, gas, rat poison, or insecticide |
| Sharp instrument/Cutting | Knife, razor, machete, or other pointed instrument (e.g., chisel or broken glass) |
| Fire/burns | Inhalation of smoke or the direct effects of fire or chemical burns |
| Fall | Jumping from a height |
| Drowning | Inhalation of liquid in bathtub, lake, or other source of water/liquid |
| Motor vehicle/Train | hit by train, car, bus, motorcycle, or other transport vehicle |
| Other (write in) |  |
| **Post from inside the US** | Use personal judgement of language and context |
| Yes |  |
| No |  |
| Cannot tell/not indicated |  |
